# Analysis of tractable allosteric sites in G protein-coupled receptors

**DOI:** 10.1038/s41598-019-42618-8

**Published:** 2019-04-16

**Authors:** Amanda E. Wakefield, Jonathan S. Mason, Sandor Vajda, György M. Keserű

**Affiliations:** 1 Department of Chemistry, Boston University, Boston, MA 02215 USA; 2 Department of Biomedical Engineering, Boston University, Boston, MA 02215 USA; 3 Sosei Heptares, Steinmetz Building, Granta Park, Great Abington, Cambridge, CB21 6DG UK; 4 Medicinal Chemistry Research Group, Research Center for Natural Sciences, Magyar tudósok krt. 2, H-1117 Budapest, Hungary

## Abstract

Allosteric modulation of G protein-coupled receptors represent a promising mechanism of pharmacological intervention. Dramatic developments witnessed in the structural biology of membrane proteins continue to reveal that the binding sites of allosteric modulators are widely distributed, including along protein surfaces. Here we restrict consideration to intrahelical and intracellular sites together with allosteric conformational locks, and show that the protein mapping tools FTMap and FTSite identify 83% and 88% of such experimentally confirmed allosteric sites within the three strongest sites found. The methods were also able to find partially hidden allosteric sites that were not fully formed in X-ray structures crystallized in the absence of allosteric ligands. These results confirm that the intrahelical sites capable of binding druglike allosteric modulators are among the strongest ligand recognition sites in a large fraction of GPCRs and suggest that both FTMap and FTSite are useful tools for identifying allosteric sites and to aid in the design of such compounds in a range of GPCR targets.

## Introduction

The vital role of G protein-coupled receptors (GPCRs) in the homeostasis and disease biology makes this class of proteins one of the most popular classes of drug targets. More than one third of the FDA approved medicines target GPCRs and the 475 drugs act at 108 unique proteins^1,2^. Major developments in the structural biology and pharmacology of GPCRs significantly facilitates the discovery of new drug candidates that is reflected in the more than 300 currently running clinical trials out of which 20% target novel GPCRs without an approved drug. Recent drug discovery efforts on GPCR targets implement emerging approaches including the development of allosteric modulators^3^, biased compounds with functional selectivity^4^, and agents acting on GPCR-signalling protein interfaces^1,5^. Due to a number of potential advantages, the development of allosteric modulators represents a popular strategy against validated GPCR targets^6^. Compared to orthosteric binders, allosteric compounds are thought to be more specific since they bind to structurally less conserved sites. Improved specificity could also be obtained by bitopic ligands bound at both the orthosteric and a nearby allosteric site. In some cases, allosteric sites might be more druggable than orthosteric ones, particularly those orthosteric sites recognizing large, peptidic ligands. Allosteric ligands might therefore represent an alternative to large endogenous ligands (peptides, lipids) with suboptimal physicochemical properties and might turn challenging targets tractable. Considering their multiple modalities, such as modulators (positive (PAM); negative (NAM); silent (SAM)), antagonist, agonist and agoPAM, and their ceiling effect, these compounds are capable to fine tune the receptor signalling and might reduce target related side-effects. In fact, there are two marketed drugs, cinacalcet (a PAM of the calcium-sensing receptor (CaSR)) and maraviroc (an allosteric antagonist of the C-C chemokine receptor 5 (CCR5))^7,8^, and multiple allosteric compounds in clinical phases.

**Figure 1:**
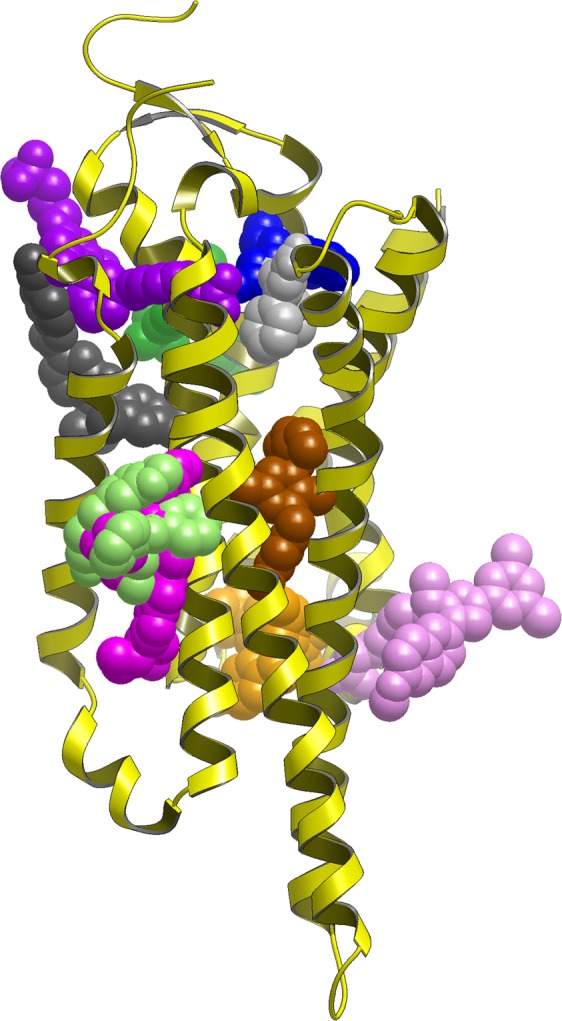
Experimentally validated allosteric sites in GPCRs. As reference shown is a Class A orthosteric antagonist ligand in grey CPK with protein in yellow ribbon (Adenosine A_2A_, triazine ligand PDB:3UZA) then from bottom to top: Intracellular Class A antagonist for CCR9 (vercinon ligand in orange CPK, PDB:5LWE); Extra-helical Class A ago-PAM for GPR40 (ligand AP8 in fuchsia CPK, PDB:5TZY); Extra-helical Class A inverse agonist for complement C5a (NDT9513727 ligand in light green CPK, PDB:5O9H); Extra-helical Class B allosteric antagonist GCGR (MK0893 ligand in pink, PDB:5EE7); Allosteric Class B antagonist CRF1 (CP376395 ligand in brown CPK, PDB:4K5Y); Extra-helical Class A antagonist for PAR2 (AZ3451 ligand in dark grey CPK, PDB:5NDZ); Allosteric Class C NAM for mGlu5 (M-MPEP in cyan CPK, PDB: Extra-helical Class A antagonist P2Y_1_ (BPTU ligand in green CPK, PDB:4XNV); Intra-helical Class A allosteric partial agonist (MK-8666 ligand in lilac, PDB:5TZR); Intra-helical Class A allosteric agonist (TAK-875 ligand in purple, PDB:4PHU); Allosteric Class A antagonist for PAR2 (AZ8838 ligand in blue CPK, PDB:5NDD).

Dramatic developments of the structural biology of membrane proteins contributes significantly to drug discovery programs. The number of available high-resolution X-ray structures now exceeds 200 representing 50 unique GPCRs. More recently, a number of cryo-electron microscopy (cryo-EM) structures contributed to structure-function studies of GPCR complexes^9^. In addition, solution phase nuclear magnetic resonance (NMR) spectroscopy in physiological conditions can complement X-ray and cryo-EM studies^10^. Allosteric ligands have been reported across the four major GPCR Classes (Classes A, B, C, and F) and the total number of allosteric X-ray structures reached 28 of 17 GPCR targets (as reported in PDB on 31^st^ May, 2018)^11^. Crystallization of GPCRs, however, is still a challenging task due to the conformational flexibility and instability of the proteins removed from the membrane. Stabilization of GPCRs could be achieved by multiple strategies that include the introduction of specific mutations (e.g. StaR® technology)^12^, stabilizing their flexible loops by fusion proteins (e.g. T4 lysozyme)^13^, or antibody fragments (nanobodies)^14^. Unfortunately, however, even these conditions do not allow crystallizing the apo proteins. Therefore, all the structures deposited in the PDB contains (i) orthosteric ligand or (ii) allosteric ligand or (iii) both. Since the structure-activity relationships for allosteric ligands are often flat or steep^15^, and small structural changes could result in mode switching^16^, structural information was found to be crucial for the identification of viable candidates. From analysing the available X-ray structures with co-crystallized allosteric ligands it became evident that allosteric sites are widely distributed (Fig. 1), including along the protein surfaces and furthermore, their plasticity and induced fit effects should be considered in drug design. Some of the allosteric sites are located in the TM bundle. These include extracellular ligand entry sites (secondary binding pockets or extracellular vestibule) that bind the orthosteric ligands temporarily upon its route to the orthosteric site, or ancestral sites that are evolutionally abandoned orthosteric sites within the transmembrane domain. Another type of allosteric site is conformational lock wherein the bound ligands can stabilise the active or inactive state of the receptor to facilitate or prevent receptor signalling. These sites can be within the hydrophobic core or located in extrahelical positions within the membrane binding region. Finally, allosteric ligands were found to interact at the intracellular signalling protein interface stabilising or preventing the binding of signalling molecules such as G proteins.

Allostery is a mechanism of regulating many biological processes such as enzyme catalysis, signal transduction, and metabolic regulation^17,18^, and hence substantial efforts have been devoted to the development of methods capable of identifying allosteric binding sites. Experimentally, Wells and co-workers developed the tethering method, and discovered an allosteric site in the caspase family^19,20^. Allosteric sites can also be detected by high-throughput screening^21,22^. Structure based approaches have been applied successfully to design allosteric inhibitors targeting transcription factors^23^ and GPCRs^11^. In addition, a variety of computational methods of binding site identification have been used for finding allosteric sites, including Allosite^24^, Fpocket^25,26^, LIGSITEcs^27^, ExProSE^28^, AlloFinder^29^, GRID^30^, SiteMap^31^, and molecular dynamics based mixed solvent methods^32^. A site detection method that has already been applied to GPCRs^33–36^ is the protein mapping tool FTMap^37,38^. FTMap (http://ftmap.bu.edu/) distributes small organic probe molecules of different size, shape, and polarity on the surface of the protein to be studied, finds the most favorable positions for each probe type, clusters the probes, and ranks the clusters based on their average energy. Regions that bind several different probe clusters are called consensus clusters or consensus sites (CSs) and predict binding hot spots. Binding hot spots are small regions on the protein surface that contribute a disproportionate amount to the ligand binding free energy, and hence ligands generally overlap with one or more hot spots^39–42^. It was shown that the consensus sites predicted by FTMap generally agree very well with the hot spots determined by a variety of experimental methods^43–49^. FTMap was expanded into a fragment-based binding site identification method called FTSite^50^. FTSite is based on the observation that the binding site of a macromolecule generally includes a strong “main” hot spot and some other secondary hot spots that are proximal and can be reached by a ligand binding the main hot spot^50^. Given the hot spots determined by FTMap, FTSite subsequently ranks the hot spots by the number of non-bonded contacts between the protein and all probes in the consensus cluster. The protein residues that are within 4 Å of the expanded hot spot form the top prediction of the binding site, defined as Site 1, while clusters of other hot spots identify lower ranked predictions. As the default, the FTSite server (http://ftsite.bu.edu/) reports the three top predicted sites. We note that both FTMap and FTSite considers only the protein structure, as all hetero atoms, including water molecules, included in the structure file are disregarded in the process of mapping.

McCammon and co-workers used FTMap for the prediction of potential allosteric sites in several GPCRs^33–36^. In their earliest work they mapped a variety of conformations of the β_1_AR and β_2_AR adrenergic receptors obtained by molecular dynamics (MD) simulations totaling approximately 0.5 μs^36^, and identified series of five potentially druggable allosteric sites for both molecules. A similar approach was later used to study the M2 muscaranic receptor^33^. Long-timescale accelerated molecular dynamics (aMD) simulations revealed distinct inactive, intermediate and active conformers of the receptor. FTMap found seven prospective allosteric binding sites, distributed in the solvent-exposed extracellular and intracellular mouth regions, as well as the lipid-exposed pockets formed by the transmembrane α-helices^33^. Recently an application of the same protocol resulted in the prediction of five non-orthosteric sites on the A_2A_ adenosine receptor^35^.

While the results by the McCammon group indicates that FTMap and FTSite can be used to detect allosteric sites of GPCRs, their analysis was restricted to four different types of targets, all belonging to the Class A group of GPCRs. Due to the recent progress in X-ray crystallography, structures are now available for many additional proteins, and here we report systematic testing of the two programs by mapping 28 structures of 17 different GPCRs covering Classes A, B, C and F. These proteins include a wide variety of allosteric binding sites across topographically distinct regions of GPCRs. In most cases the structures are of the GPCR protein co-crystallized with an allosteric ligand. Using these structures, we tested whether FTMap and FTSite are able to identify these preformed allosteric pockets as top scoring binding sites (retrospective validation). There are, however, pairs of structures with liganded and unliganded allosteric sites that allowed us to predict the allosteric binding pockets prospectively. Some of the sites are partially hidden and are not fully formed in crystal structures without a bound allosteric ligand. Our motivations substantially differ from those of the previous studies. First, while McCammon and co-workers used MD simulations to generate conformational ensembles to predict potential novel allosteric sites, here we study how reliably FTMap can identify the known sites that bind allosteric ligands. This question is far from trivial, because most GPCRs have a variety of sites that bind orthosteric modulators, lipids, and possibly a variety of crystallization additives. Thus, it is important to determine the ranking of the allosteric site among all these various pockets. Second, we also study how strong these sites are, as the strength of the hot spots relates to their druggability^51^. Third, FTMap has been developed for mapping soluble globular proteins. Apart from the work by the McCammon group on four GPCRs, the only transmembrane protein mapped by the program was the influenza M2 proton channel^46,52^. Although we succeeded in capturing the potential inhibitor binding sites both inside and outside of the four-helix bundle of the channel, the general applicability of the method to GPCRs was questionable. As will be discussed, the ability of the program of detecting intrahelical allosteric sites confirms that the region is likely to be well solvated. However, the druggability criteria developed for soluble proteins may not fully apply, indicating potential differences in the mechanism of ligand recognition.

## Results and Discussion

### Retrospective analysis of allosteric sites

Crystal structures of GPCRs complexed with small molecule allosteric modulators were collected from the PDB^53^. The 28 structures available at the time of our analysis (May 2018) cover four classes including 15 Class A, 5 Class B, 6 Class C and 3 Class F GPCRs (Table 1). Experimentally validated allosteric sites were assigned by their type (intrahelical – HC, conformational lock – CL, signalling interface – SI) and location (extracellular side – EC, helical bundle – TM, intracellular side – IC). Next, we used FTMap and FTSite to explore the potential binding sites using the pseudo-apo structures generated after removing the small molecule modulator (Table 2). In these cases, our objective was testing whether FTMap and FTSite are able to identify the preformed allosteric pocket within the top scoring binding sites. For each structure mapped, Table 2 shows the number of consensus sites within 5 Å of the allosteric site and lists the sites with the number of probe clusters at each site indicated in parenthesis. The consensus sites are ranked on the basis of the number of probe clusters contained. Accordingly, the FTMap rank in Table 2 indicates the highest rank of any consensus site (hot spot) located at the allosteric site. Based on the notation established in the FTMap server (http://ftmap.bu.edu), the consensus sites are numbered starting from 0, with the number of probe clusters at the consensus site shown in parenthesis.

**Table 1:** High resolution X-ray structures of GPCRs co-crystallized with small molecule allosteric ligands.

Class	Target	Ligand code	Ligand name	PDB	Site Type	Site Location
A	BETA2	8VS	CMPD-15PA	5X7D	SI	IC
A	C5A	9P2	NDT9513727	5O9H	CL	EH
A	CCR2	VT5	CCR2-RA-[R]	5T1A	SI	IC
A	CCR5	MRV	Maraviroc	4MBS	HC	TM
A	CCR9	79 K	Vercirnon	5LWE	SI	IC
B	CRF1R	1Q5	CP-376395	4K5Y	CL	TM (IC)
A	CXCR4	ITD	IT1t	3ODU	HC	TM
A	CXCR4	PRD	CVX15	3OE0	HC	EC-TM
B	GLP1	97Y	PF-0637222	5VEW	SI	EH-IC
B	GLP1	97 V	NNC0640	5VEX	SI	EH-IC
B	GCGR	5MV	MK-0893	5EE7	CL	EH-IC
B	GCGR	97 V	NNC0640	5XEZ	CL	EH-IC
A	GPR40	2YB	TAK-875	4PHU	CL	EH-EC-TM
A	GPR40	7OS	MK-8666	5TZR	CL	EH-EC-TM
A	GPR40	7OS	AP8	5TZY	CL	EH-IC
A	M2	2CU	LY2119620	4MQT	HC	TM-EC
A	P2Y1	BUR	BPTU	4XNV	CL	EH-EC
C	MGLU1	FM9	FITM	4OR2	HC	TM
C	MGLU5	2U8	Mavoglurant	4OO9	HC	TM
C	MGLU5	51D	CMPD-25	5CGC	HC	TM
C	MGLU5	51E	HTL14242	5CGD	HC	TM
C	MGLU5	D8B	M-MPEP	6FFI	HC	TM
C	MGLU5	D7W	Fenobam	6FFH	HC	TM
A	PAR2	8TZ	AZ8838	5NDD	HC/CL	TM
A	PAR2	8UN	AZ3451	5NDZ	HC/CL	TM
F	SMO	CLR	Cholesterol	5L7D	HC/CL	EC
F	SMO	VIS	Vismodegib	5L7I	HC/CL	EC-TM
F	SMO	SNT	SANT-1	4N4W	HC/CL	EC-TM

Site types are assigned as intrahelical – HC, conformational lock – CL, signaling interface – SI. Site location is indicated as intra-helical bundle – TM, extra-helical – EH, extracellular side – EC, intracellular side – IC.

**Table 2:** FTMap and FTSite results obtained for GPCR structures co-crystallized with allosteric ligands.

PDB	Target	# FTMap Clusters	FTMap Clusters within 5 Å of the allosteric site	FTMap Rank	FTSite Rank
5X7D	BETA2	7	0(18), 5(7)	1	2
5O9H	C5A	8	2(12)	3*	—
5T1A	CCR2	7	1(16), 2(15), 4(8)	2	3
4MBS	CCR5	7	0(19), 1(16), 2(15), 4(9), 5(9)	1	1
5LWE	CCR9	7	1(13), 2(13), 4(11), 5(6)	2	1
4K5Y	CRF1R	8	1(11), 3(10), 5(7), 6(6)	2*	2*
3ODU	CXCR4	9	0(22), 1(14), 2(12), 3(10), 5(6), 6(5), 7(5)	1	1
3OE0	CXCR4	10	0(17), 3(8), 4(8), 5(6), 6(6), 7(5)	1	1
5VEW	GLP1	6	NA	—	—
5VEX	GLP1	7	5(5)	6*	3*
5EE7	GCGR	8	NA	—	—
5XEZ	GCGR	—	NA	—	—
4PHU	GPR40	7	2(13), 3(10)	3*	3*
5TZY	GPR40	10	0(16), 3(9), 9(5)	1*	3*
5TZR	GPR40	6	0(20), 5(10)	1*	1*
4MQT	M2	9	1(14), 2(12), 4(7)	2	1
4OR2	MGLU1	9	1(13), 2(10), 3(8), 4(7), 5(7), 6(6)	2	1
4OO9	MGLU5	9	1(11), 4(6)	2	1
5CGC	MGLU5	7	5(7)	6	3
5CGD	MGLU5	8	3(8), 7(5)	4	3
6FFI	MGLU5	7	1(17), 5(5)	2*	—
6FFH	MGLU5	8	3(10), 7(5)	4*	—
4XNV	P2Y1	7	NA	—	—
5NDD	PAR2	9	0(17), 1(11)	1*	2
5NDZ	PAR2	10	0(17)	1*	3
5L7D	SMO	8	0(26), 3(13), 5(7)	1*	1*
5L7I	SMO	8	0(16), 1(16), 2(11), 3(11), 6(7)	1	1*
4N4W	SMO	8	0(20), 4(8), 6(6)	1	2

*Indicates that domain splitting was used.

**Figure 2:**
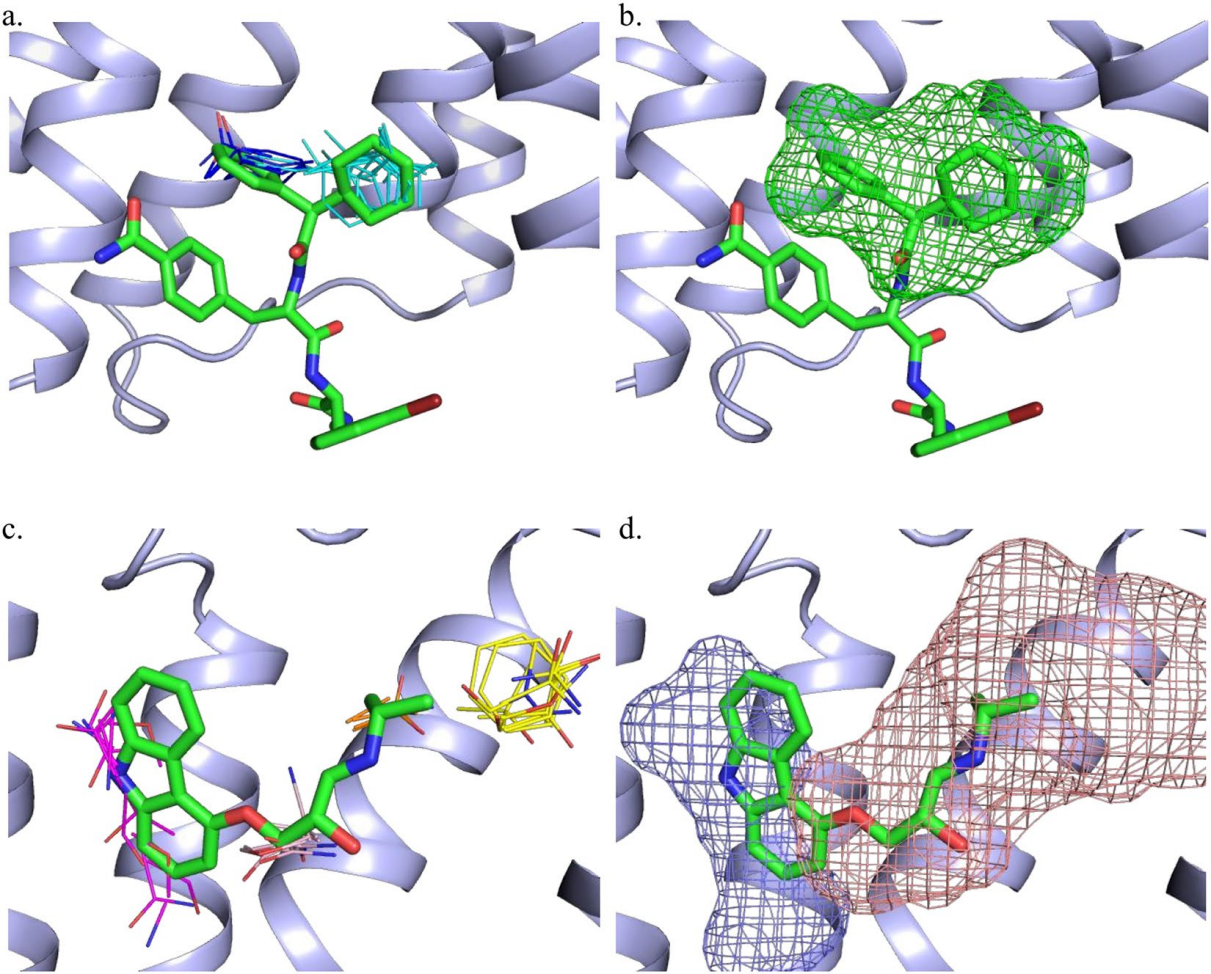
Hot spots and allosteric ligand binding sites predicted by (**a**) FTMap and (**b**) FTSite for PDB 5X7D. Also shown are the hot spots and orthosteric ligand binding site by (**c**) FTMap and (**d**) FTSite. We note that in this and all following figures, each probe cluster is represented by the structure of a single probe at the cluster center. Both ligands are represented by green sticks. The FTMap hot spots, shown as lines, are coloured by rank in the following order: cyan, hot pink, yellow, light pink, white, blue and orange. The FTSite sites, shown as mesh, are coloured, by rank, in the following order: pink, green, and purple.

For example, results for the structure 5X7D at the top of Table 2 reveal that the allosteric site of BETA2 within 5 Å of the allosteric modulator 8VS (see Table 1) includes the strongest consensus site 0(18) formed by 18 probe clusters, and also the 6^th^ strongest consensus site 5(7) formed by 7 probe clusters. Since the allosteric site includes the strongest consensus site, its FTMap rank is 1 (Fig. 2). As mentioned in the Introduction, FTSite ranks the predicted binding sites on the basis of the total number of contacts between the protein and all probes within a specific site, and using this definition the allosteric site in 5X7D has the FTSite rank 2 rather than 1 (Table 2). Thus, FTMap and FTSite measure somewhat different properties. The two results show that in 5X7D the allosteric site has the strongest hot spot (consensus site), indicating a surface patch with high level of binding propensity, which was shown to relate to druggability^51^. However, based on FTSite, which measures the total number of probes binding in a region that generally includes several adjacent hot spots, there is a site that has more probes than the allosteric site. This site with the FTSite rank 1 (see Table 2) is formed by the consensus clusters 1(14), 2(13), 3(10), and 6(5), and it binds the orthosteric antagonist carazolol. Thus, these results show a competition between allosteric and orthosteric sites for the binding of non-specific probes, and indicate that the allosteric site presents the strongest hot spot with the highest density of bound molecular probes, in spite of the existence of a strong orthosteric site in the same structure. In the following we discuss the results, shown in Table 2, for the various types of allosteric sites.

#### Intrahelical allosteric sites

These sites are located between the transmembrane helices. We have divided our intrahelical allosteric sites into two subclasses as ligand entry and ancestral sites. The only target showing the allosteric ligand entry site is M2^54^. For this receptor FTMap found 9 significantly populated consensus clusters out of which 1(14), 2(12), and 4(7) were found to be overlapped with the allosteric binding site, and formed the highest ranked FTSite site. Interestingly the strongest consensus cluster, 0(15), was located at the other end of the transmembrane domain, at the site that binds a nanobody in the X-ray structure 4MQS. The consensus clusters 5(6) and 6(5) were located at the site that binds the orthosteric agonist iperoxo in both structures.

**Figure 3:**
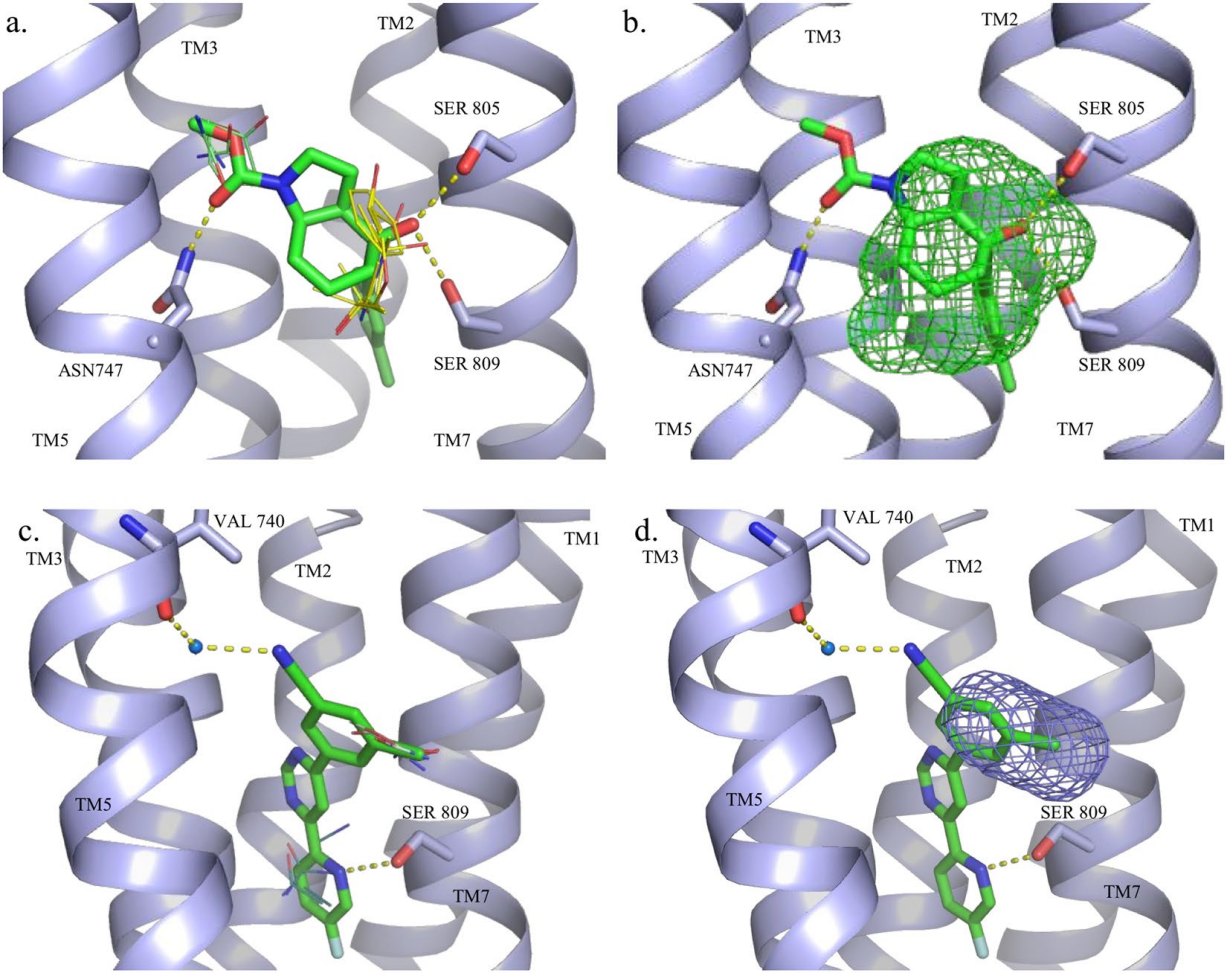
Hot spots and ligand binding sites predicted, respectively, by (**a**) FTMap and (**b**) FTSite for the mGluR5-mavoglurant structure (PDB: 4OO9) and by (**c**) FTMap and (**d**) FTSite for the mGluR5-HTL14242 structure (PDB: 5CGD). The allosteric ligand mavoglurant is represented by green sticks. The FTMap hot spots are shown as lines, 1(11) in yellow and 4(6) in green. The second ranked site, predicted by FTSite, is shown as green mesh. The allosteric ligand HTL14242 is represented by green sticks. HOH4115 is represented by a blue sphere. The FTMap hot spots, shown as lines, are colored as follows: 3(8) in white and 7(5) in teal. The third ranked FTSite site is shown as purple mesh.

The other subclass of intrahelical sites are considered ancestral and exemplified by CXCR4 (2 structures)^55^, CCR5 (1 structure)^56^, mGluR1 (1 structure)^57^, mGluR5 (5 structures)^58–60^, SMO (3 structures)^61,62^, and PAR2 (2 structures)^63^. For the chemokine receptors (CXCR4 and CCR5), SMO and PAR2 the allosteric site is located at the extracellular side close to the ligand entry site while the ancestral site in mGlu receptors is located deeper in the helical bundle. Out of the eleven structures with ancestral intrahelical allosteric sites, nine of the structures’ ligands were predicted by one of the top three ranked FTMap consensus sites. FTMap worked extremely well for the chemokine structures and resulted in a high number of top ranked consensus clusters overlapping with the allosteric binding site. For CXCR4 structures, the large majority of consensus clusters, including the strongest ones, were in close proximity of the crystallographic ligand pose. This finding, together with the large number of probe clusters in these consensus clusters indicates that the allosteric site is a very strong binding site. Although FTMap showed similar performance on two of the SMO structures 5L7I and 4N4W, for the other structure (5L7D) that contains a different ligand, it found only the third and fifth ranked consensus clusters 2(12) and 4(10) at the allosteric pocket. Nevertheless, the two consensus clusters resulted in the binding site top ranked by FTSite (Table 2). We note that FTMap placed the strongest hot spot 0(23) at the intrahelical allosteric site that binds the antagonist SNT in the analogue 4N4W. In both structures 5NDD and 5NDZ of the PAR2 receptor FTMap placed the strongest consensus clusters at the allosteric site. FTMap predicted the allosteric site at the mGluR1 with high confidence (Table 2). Interestingly, the two lower ranked sites (4^th^ and 6^th^) both overlapped with similar ligands from mGluR5 structures. We have a high number of X-ray structures available for mGluR5, Here 4OO9 contains mavoglurant that represents the classical acetylenic negative allosteric modulators^59^, while in 5CGD there is a tricyclic structure (HTL14242) co-crystallized^58^. FTMap identified 9 significant consensus clusters in the mavoglurant structure, out of which 1(11) and 4(6) were found to be overlapped with the position of the ligand (Fig. 3a). If we combine the hot spot 4(6) with the adjacent consensus clusters 10(3) and 11(3), then we get a consensus cluster with 10 probe clusters that now ranks second instead of fourth. Combining all consensus clusters within 5 Å of the ligands we get a consensus cluster with 21 probe clusters, thus representing the highest ranked hot spot. In line with this observation, FTSite was able to correctly identify the allosteric binding site as the top ranked site (Fig. 3b).

In contrast to mavoglurant, ligands in 5CGD and 5CGC do not contain the acetylenic linker and the induced fit effects make the overall shape of the ligands markedly different^58^. For 5CGD, FTMap found eight consensus clusters, two of which, 3(8) and 7(5), overlapped with the allosteric ligand (Fig. 3c). These clusters were also less populated and were ranked 4^th^ however, the combination of the two clusters would increase the ranking of the site to the third strongest. Like the 4OO9 structure, FTSite provided better result ranking the experimental allosteric site being the third most significant (Fig. 3d). Mapping 5CGC^58^ with an analogue of HTL14242 we obtained similar result. For 5CGC FTMap found one consensus site to be overlapped with the ligand. FTSite’s third ranked site predicted the allosteric binding site. This cluster was found to be less populated and were ranked 6^th^ out of the seven clusters identified (see Supplementary Fig. S1).

The most recent mGluR5 structures co-crystallized with M-MPEP and fenobam (6FFI and 6FFH) show similar helical organization as seen with other ligands^60^. The location and general architecture of the allosteric pocket closely resembles to the Heptares structures (5CGC and 5CGD).For 6FFI, FTMap identified 8 consensus clusters out of which two, 1(17) and 5(5), were located at the allosteric site (see Supplementary Fig. S2). When combined, the two consensus clusters within the allosteric binding site ranked as the top site. These results were similar to the mavoglurant structure. For the fenobam bound structure (6FFH) FTMap predicted 7 consensus clusters two of which, 3(10) and 7(5), were found in the allosteric pocket (see Supplementary Fig. S3). FTSite was unable to detect the deep allosteric site, but FTSite’s top ranked sites made up a large, intrahelical site close to the intrahelical side.

#### Allosteric conformational locks

In contrast to compounds recognized by intrahelical sites at or adjacent to the orthosteric ligands, allosteric ligands might bind to other sites that contribute to the stabilization of the active (positive modulator, agonist) or the inactive (negative modulator, antagonist) conformational state of the GPCR and therefore changing receptor signalling. Targets with this mechanism of action are CRF1^64^, P2Y1 cocrystallized with BPTU^65^, C5A^66^ (one structure for each), GCGR^67,68^ (2 structures), and GPR40^69,70^ (3 structures). These crystal structures with bound allosteric ligands that were subjected to FTMap and FTSite analysis. Both methods were able to predict four out of the six allosteric binding sites in the bound structures. FTMap identified several hot spots for each of the receptors and for CRF1R, C5a and GPR40 the allosteric site was ranked mostly between the first three predicted binding site. The highest number of overlapping consensus clusters was observed for CRF1 (4 out of 8), it was reasonably large for the GPR40 structures (2 of 7, 3 of 10 and 2 of 6) and lower for C5a (1 of 8). Most importantly, however, at least one of the overlapping consensus clusters showed reasonably high numbers of probe clusters that confirmed the strength of the allosteric site. Both FTMap and FTSite failed to predict the binding sites for GCGR and P2Y1 that are located at the external surface of the receptor. As already mentioned, the present version of FTMap and FTSite is not parameterized for lipids and therefore could not predict sites at the receptor-membrane interface.

**Figure 4:**
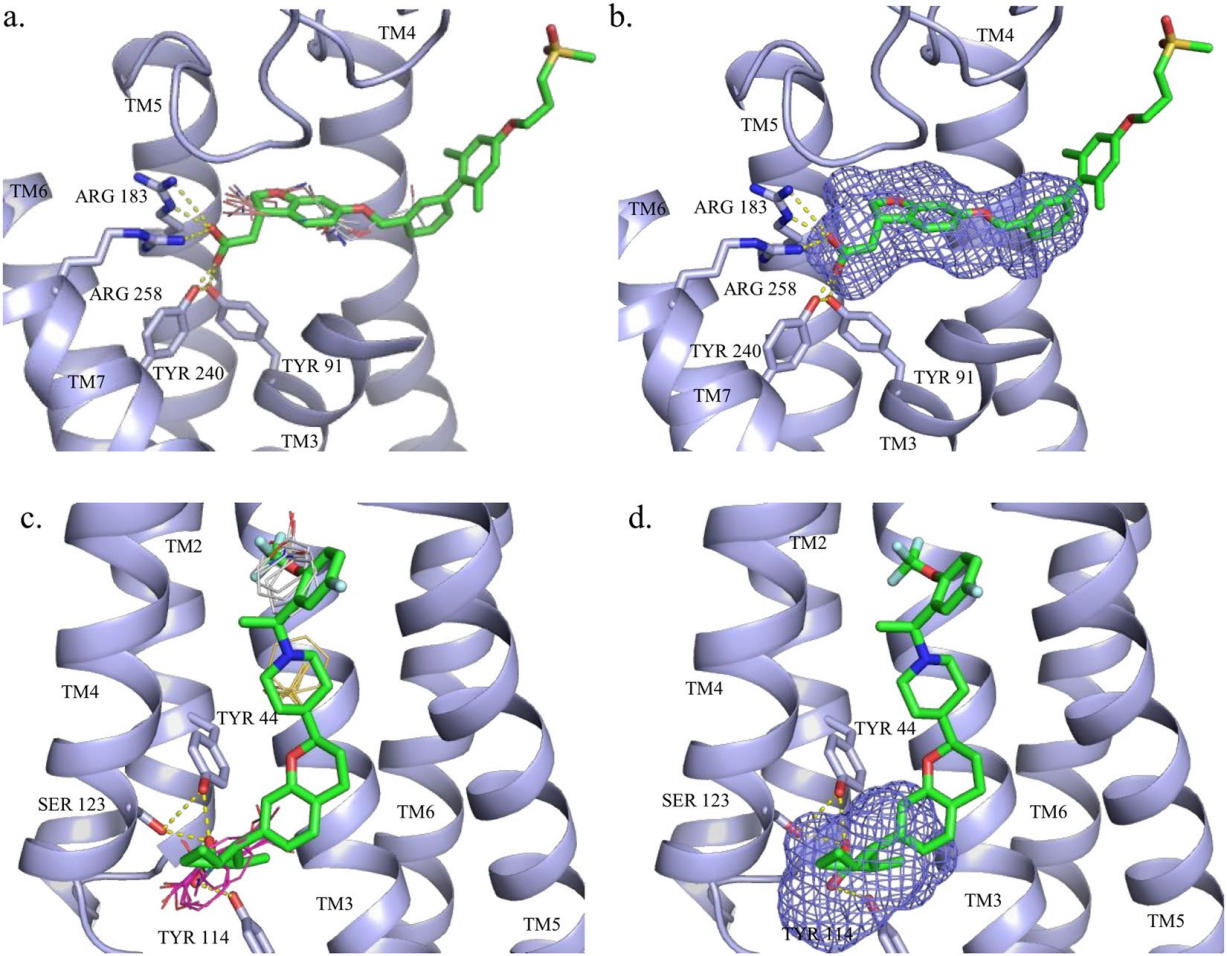
Hot spots and ligand binding sites predicted, respectively, by (**a**) FTMap and (**b**) FTSite for the GPR40-TAK-875 structure (PDB: 4PHU) and by (**c**) FTMap and (**d**) FTSite for the GPR40-AP8 structure (PDB: 5TZY). The allosteric ligand TAK-875 is represented by green sticks. The FTMap hot spots, shown as lines, are 2(13) in light pink and 3(10) in white. The third ranked FTSite site is shown as purple mesh. The allosteric ligand AP8 is represented by green sticks. The FTMap hot spots, shown as lines are 0(16) in pink, 3(9) in white and 9(5) in yellow. The third ranked FTSite site is shown as purple mesh.

GPR40 has three complexes available in the PDB. FTMap analysis of 4PHU^69^ identified 7 consensus sites out of which the adjacent consensus clusters 3(9) and 4(9) were located deep in the allosteric pocket (Fig. 4a). FTMap’s strongest cluster corresponded to the binding site of 1-Oleoyl-R-glycerol. However, the centers of the consensus clusters 3(9) and 4(9) are less than 5 Å from each other, and combining the two clusters would yield the strongest hot spot. Similarly to FTMap, FTSite predicted the binding site of a lipid component, 1-oleoyl-R-glycerol, as Site 1, and the allosteric site as Site 3. FTSite’s second ranked site, Site 2, overlaps with the binding site (Fig. 4b). Please note, however, that a significant part of the ligand is positioned outside the helical bundle and forms direct interactions with membrane lipids. FTMap and FTSite could not deal with this part of the binding pocket.

In the GPR40-MK-8666 structure 5TZR FTMap predicted 6 consensus sites altogether out of which two were identified within the allosteric binding site with particularly high probe numbers. The allosteric site was predicted by both FTMap and FTSite as the top ranked site.

Application of FTMap to the GPR40 structure 5TZY yielded 10 consensus clusters, with 0(16), 3(9), and 9(5) located in the allosteric site, thus including the strongest hot spot (Fig. 4c). In spite of the very strong hot spots in the allosteric site, FTSite ranked the experimental allosteric pocket as Site 3 (see Fig. 4d). In contrast to TAK-875 (PDB: 4PHU) and MK-8666 (PDB: 5TZR), AP8 is a full allosteric agonist (AgoPAM) of GPR40 (PDB:5TZY) and its allosteric binding site is completely different from that found for the partial agonists. The AP8 binding site is formed by helices II–V and ICL2^70^. The carboxylate group of the ligand forms hydrogen bonds Tyr44, Ser123 and Tyr114. The cyclopropyl group is accommodated in a hydrophobic pocket of Leu106, Tyr114, Phe117, and Tyr122. The chroman core forms hydrophobic interactions with Ala99, Ala102, Val126 and Ile197, while the terminal trifluoromethoxyphenyl ring is surrounded in a hydrophobic cavity with Ile130, Leu133, Val134, Leu190, and Leu193.

#### Intracellular allosteric sites

Although the popularity of GPCR targets was typically associated to their tractable deep extracellular binding sites, recent results highlighted that targeting them from the intracellular side is also feasible. Crystal structures with allosteric modulators revealed that the intracellular signalling surface of GPCRs is available for small molecule binding with a potential of modulating the receptor function and signalling. The targets for intracellular allosteric sites included CCR2^71^, BETA2^72^, CCR9^73^ (one structure for each) and GLP1 with two structures^74^. FTMap accurately predicted the allosteric binding sites in all targets except for the two GLP1 structures. In 5VEW (GLP1), there is a modified cystine (S-(2-amino-2-oxoethyl)-l-cysteine) that restricts the movement of the intracellular tip of helix VI. FTMap predicts the site of this modified residue as the third strongest binding site 2(16) within the protein. The mutated residue was changed back to a cysteine for the purpose of mapping in both GLP1 structures. The second GLP1 structure, 5VEX, has a weak hot spot 5(5) that overlaps with the allosteric site. However, the modified cysteine residue overlaps with the fourth ranked cluster 3(12). In 5VEW, FTSite’s top ranked site identified the modified residue, CSD. The FTSite results from the second GLP1 structure, 5VEX, show the third ranked binding site around the CSD region.

**Figure 5:**
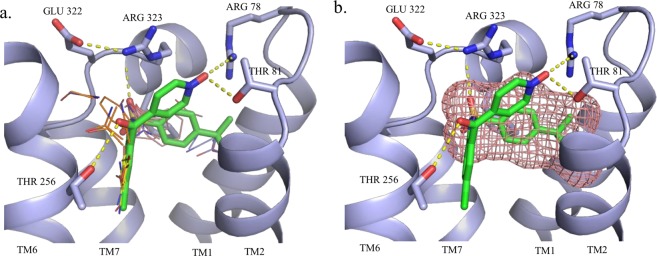
Hot spots and ligand binding sites predicted, respectively, by (**a**) FTMap and (**b**) FTSite for the CCR9-vercirnon structure (PDB: 5LWE). The allosteric ligand vercirnon is represented by green sticks. The FTMap hot spots, shown as lines, 1(13) in yellow, 2(13) in light pink, 4(11) in blue and 5(6) in orange. The highest ranked FTSite site is shown by pink mesh.

In the case of CCR2, FTMap identified 7 hot spots out of which 3 were overlapped with the allosteric site that was ranked 2^nd^. In this case the top ranked hot spot overlapped with the orthosteric binding site of 73 R. For the BETA2 structure (5X7D), FTMap predicted 7 sites in total and two of these were overlapped with the experimental binding site ranked first. FTSite was not able to improve these predictions and provided the site at the third and the second place of its ranked list. For the CCR9-vercirnon complex (5LWE)^73^ FTMap predicted 7 consensus sites, and 1(13), 2(13, and 4(11), and 5(6) were found in the experimentally validated allosteric pocket (Fig. 5a). These results indicated a large pocket at the allosteric site. The top ranked hot spot 0(15) overlapped with the binding site of a lipid component, 1-oleoyl-R-glycerol, but this hot spot was isolated. In contrast, the four hot spots in the allosteric site are close to each other, and it becomes the top ranked site when the adjacent hot spots are combined. Accordingly, FTSite identified the allosteric site as its top ranked predicted site (Fig. 5b).

#### Prospective identification of allosteric sites

Hidden and partially hidden allosteric sites are invisible or only partly visible in X-ray structures crystallized in the absence of allosteric ligands. These sites therefore represent a true challenge for prediction algorithm and are well suited to investigate the performance of FTMap and FTSite. There are four pairs of GPCR structures available in the PDB for muscarinic M2, adrenergic *β*2, GPR40 and P2Y1 receptors (Table 3). In each pair, the first structure binds only an orthosteric ligand, and the second binds both the same orthosteric one and an allosteric ligand. We were specifically interested whether FTMap and FTSite would be able to predict the experimentally validated allosteric sites based on the structure of the complex with only orthosteric ligand, and hence mapped both structures.

**Table 3:** FTMap and FTSite results obtained for the orthosteric and allosteric pairs of GPCR complexes.

Target	Ligand type	Ligand name	PDB	# FTMap Clusters	FTMap Clusters w/5A	FTMap allosteric rank	FTSite allosteric rank
BETA2	orthosteric	Carazolol	2RH1	10	**1(12)**	2*	3*
BETA2	allosteric	Carazolol and Cmpd-15PA	5X7D	7	**0(18), 5(7)**	1	2
M2	orthosteric	Iperoxo	4MQS	10	**0(19), 6(8)**	1*	1*
M2	allosteric	Iperoxo and LY2119620	4MQT	9	**1(14), 2(12), 4(7)**	2*	1*
GPR40	orthosteric	MK-8666	5TZR	6	**0(20), 5(10)**	1*	2*
GPR40	allosteric	MK-8666 and AP8	5TZY	10	**0(16), 3(9), 9(5)**	1*	3*
P2Y1	orthosteric	MRS2500	4XNW	5	—	—	—
P2Y1	allosteric	BPTU	4XNV	7	—	—	—

*Indicates that domain splitting was used.

Since allosteric binding is usually accompanied with conformational changes, orthosteric and allosteric pairs were first subjected to comparative binding site analysis using Fpocket^25,26^. First, we used Fpocket for the characterisation of binding pockets and analysing conformational changes around the orthosteric and allosteric pockets (see details in the Supplementary Table S1). Next, we used FTMap and FTSite on the orthosteric structures to predict the allosteric site confirmed by the corresponding allosteric structure.

#### β2 adrenergic receptor

Comparative Fpocket analysis revealed that there are significant structural changes between the bound^72^ and unbound^75^ allosteric site pocket. Phe332, Phe336 and Arg63 are pushed out of the pocket to make room for the allosteric ligand. Asp331 also is shifted a bit out of the pocket so that it can form interactions with Lys267 that moves in to form favorable interaction with Asp331. The orthosteric site has very minor changes between the structures. The pocket volume of the orthosteric site and the druggability score decrease when the allosteric ligand is present (Supplementary Table S1). The allosteric site’s volume and druggability score, however increase upon binding of the allosteric ligand.

**Figure 6:**
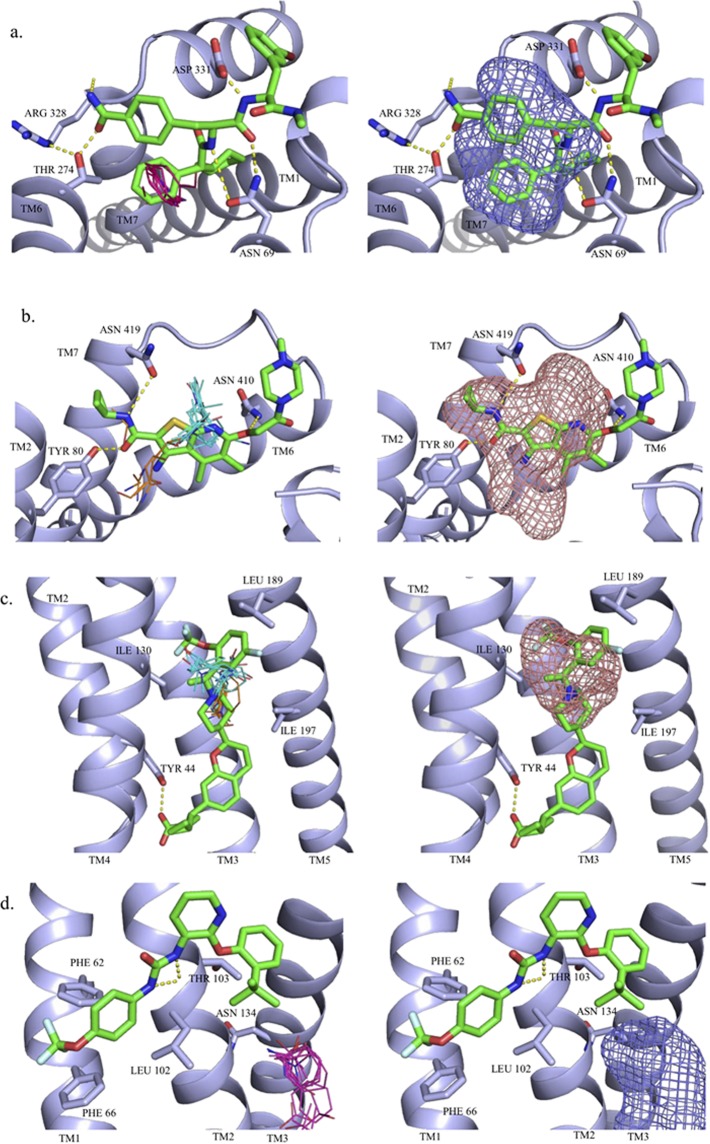
Hot spots and ligand binding sites predicted by FTMap and by FTSite for the orthosteric complexes of (**a**) beta2 (PDB:2RH1), (**b**) M2 (PDB:4MQS), (**c**) GPR40 (PDB:5TZR) and (**d**) P2Y1 (PDB:4XNW) receptors. The allosteric ligands are represented by green sticks. The FTMap hot spots, shown as lines, are coloured are coloured by rank in the following order: cyan, hot pink, yellow, light pink, white, blue and orange. The FTSite sites, shown as mesh, are coloured, by rank, in the following order: pink, green, and purple.

Mapping results obtained for the X-ray structure 5X7D binding both the orthosteric antagonist carazolol and the intracellular allosteric antagonist compound-15PA^72^ were already discussed and we compared these to binding hot spots identified by FTMap and FTSite for the orthosteric carazolol-only structure (2RH1). FTMap was able to identify the allosteric site partially hidden in this structure with its second ranked consensus site 1(12). FTSite was unable to predict the allosteric site in the unbound orthosteric complex. In fact, the FTMap results for 2RH1 reveals that the orthosteric site is extremely strong, and includes the hot spots 0(18), 2(11), 3(10), 4(9), 5(8), and 6(6), and FTSite places all three predicted site at this location (Fig. 6a). In spite of the very strong orthosteric site, the mapping of the structure without any allosteric ligands still identifies the allosteric site as the second strongest hot spot.

#### Muscarinic M2 receptor

Comparing the orthosteric and allosteric structures of the M2 receptor revealed only minor changes at both sites. At the allosteric only Trp422 rotates to have its ring structures align in parallel with the rings in the allosteric ligand. At the orthosteric site we found very minor changes in side chain orientations. In the allosteric structure (4MQT) Fpocket detected only one combined binding site filled by the orthosteric and the allosteric ligands. Comparing orthosteric and allosteric pocket volumes calculated for both of the structures showed that no significant new pocket was formed upon the binding of the allosteric ligand. Interestingly, however, the druggability of the combined allosteric pocket has been increased significantly (Supplementary Table S1).

Mapping results obtained for the agonist bound iperoxo structure (4MQS)^54^ were compared to the previously described PAM complex of LY2119620 (4MQT)^54^ that had both the allosteric modulator and iperoxo. As shown in Table 3, both FTMap and FTSite predicted the hidden allosteric site in the orthosteric complex as the top ranked site (Fig. 6b). Interestingly, mapping the structure 4QMS without an allosteric ligand the allosteric site had a stronger hot spot, 0(19), than mapping 4MQT that had bound ligands at both orthosteric and allosteric sites. This confirm the presence of a well-formed strong allosteric site.

#### Free fatty acid receptor 1 (GPR40)

The other pair of homologs studied consisted of the GPR40 structure with the partial agonist MK-8666 and the positive allosteric modulator AP8 with agonist activity (5TZY), and the GPR40 structure co-crystallized only with the orthosteric ligand MK-8666 (5TZR)^70^. Fpocket analysis of the orthosteric and allosteric structures revealed that the allosteric site has a pocket that opens up slightly to accommodate the allosteric ligand. Pro40, Ile130 and Leu190 moving out of the pocket while Ser123 moves into the pocket to form polar interactions with the ligand. Comparing the orthosteric sites we found that the ligand (MK6) adopts a slightly different orientation especially around the sulfate functional group when the allosteric modulator is bound. LEU 158 moves into the site which causes MK6 to shift slightly outwards. Pocket volumes of the orthosteric and allosteric sites increase upon the allosteric ligand binding. The druggability score of the orthosteric site decreases while it is increased for the allosteric site upon binding of the allosteric ligand (Supplementary Table S1).

The allosteric sites of 5TZY and its unbound homolog, 5TZR, were predicted by FTMap’s top ranked sites (Fig. 6c). Again, mapping the structure 5TZR without an allosteric ligand placed a stronger hot spot, 0(20), at the hidden allosteric site than mapping the structure 5TZY with both allosteric and orthosteric ligands. FTSite ranked the allosteric site second in 5TZR and third in 5TZY. The predicted sites are in a large open pocket near the membrane - intercellular interface. An additional pair of structures of the P2Y1 receptor with an orthosteric and an allosteric ligands proved a challenge to FTMap, as the allosteric site was extrahelical in the membrane binding region.

#### Purinergic P2Y1 receptor

In the case of this receptor, we found that the allosteric pocket tightens up around the bound allosteric ligand. Phe119 moves into the binding site to form hydrophobic interactions with the ligand. Leu102 sidechain flips slightly away from the pocket to form more room for the ligand. Interestingly, Fpocket was unable to detect the allosteric binding site in the bound conformation. Although the identification of the preformed allosteric site seems to be trivial, this failure highlights the importance of retrospective validation. Comparing the orthosteric sites we found the unbound site much more open. Lys41 and Arg287 shift out of the binding site to accommodate the ligand. Leu44 sidechain shifts into the pocket to form hydrophobic interactions with the ligand while Gln40, Lys46, Arg195 and Tyr110 shift into the pocket to form polar interactions with the phosphate group. The pocket volume and the druggability score of the orthosteric site increase in the bound structure (Supplementary Table S1).

In the orthosteric 4XNW structure, the ligand MRS2500 is bound within the seven transmembrane bundle. FTMap was able to predict the MRS2500 binding site with its top and third ranked hot spots 0(21) and 2(16). These hot spots were very strong and indicate that this is a druggable site. Indeed, MRS2500 has the K_i_ value of 0.8 nM. FTSite’s top ranked site also predicted the binding site of MRS2500. Notice that the mapping of the allosteric complex 4XNV of P2Y1R finds the same strong site that binds MRS2500, although in 4XNV the protein is co-crystallized with the non-nucleotide antagonist BPTU, which binds to an allosteric pocket on the external receptor interface with the lipid bilayer, entirely outside of the helical bundle. Note that FTMap failed whether or not the structure was co-crystallized with the allosteric ligand. The orthosteric sites held the majority of top ranked consensus sites, which indicates that the orthosteric site is much stronger than the allosteric site (Fig. 6d). FTSite’s first and second ranked sites aligned with the orthosteric sites for 4XNV^65^. The third site was in the protein-membrane interface where a cholesterol hemisuccinate molecule was bound. For 4XNW^65^, FTSite’s first and third sites overlapped with the orthosteric site and the second site was within the protein-membrane interface. While the failure to predict the BPTU allosteric site was disappointing, it can be explained by the already discussed limitations of the of the mapping tools that have been parameterized to find hot spots and binding sites of globular proteins. Prediction of this allosteric site based on the orthosteric structure is even more challenging as the site is induced by the ligand and hardly visible in the structure with the orthosteric ligand. However, it was very exciting that FTMap was able to predict all intra-helical allosteric sites even in the absence of allosteric ligands in the crystal structures.

We emphasize that the structures 2RH1 of BETA2, 4MQS of M2, and 5TZR of GPR40 have been determined without a bound allosteric ligand, and yet FTMap placed the strongest or second strongest hot spot at the allosteric site. While these predictions are not genuinely prospective, the FTMap server has been publicly available since 2009 and has been applied to these structures without any adjustment in the algorithm or the parameters. Thus, the results were not affected by the fact that the inhibitor-bound structures were known. However, we must also note that false positives may occur when the strongest hot spot is not located at the allosteric site. In some structures such strong hot spots identify the orthosteric site (e.g., in 5T1A, 5LWE, and 4PHU). In contrast, the allosteric site is only the second or third strongest hot spot in 5O9H, 4K5Y, and 6FFI, and none of the strong hot spots are at the allosteric site in the GLP1 and GCGR structures, as well as in most structures of MGLU5.

## Conclusion

Here we report the performance of two mapping algorithms, FTMap and FTSite on the prediction of allosteric sites in GPCRs. Investigating the wide range of allosteric sites identified at different locations in the 29 crystal structures of 17 receptors from four classes of GPCR targets we showed that both algorithms ranked the experimental allosteric site mostly within their top three sites. These predictions represent 69% and 76% of the cases for FTMap and FTSite respectively. The predictions failed only for sites located at the protein-membrane interface. This limitation is not surprising since the mapping tools have been developed for predict binding hot spots and binding sites of soluble globular proteins. It will require substantial further work, involving membrane modelling and major reparameterization of the interaction potential to enable the methods to also identify membrane-protein interface sites. Representing the membrane as a low dielectric region improved mapping results for the influenza M2 proton channel^46,52^. However, using this simple model was not sufficient to find inhibitor binding sites located at the protein-membrane interface. In fact, the inhibitors are generally large and include both several hydrophobic and hydrogen bonding groups. Thus, it is very likely that a version of FTMap specifically developed for the identification of hot spots at the protein-membrane interface will also require a different set of molecular probes, in addition to a more detailed model of the membrane.

Considering only the sites located within the protein interior, the success rates by FTMap and FTSite, respectively, reach 80% and 88%. It is important also to note that not considering ligand binding in the membrane, FTMap and FTSite were able to predict partially hidden allosteric sites (see adrenergic *β*2 (2RH1), muscarinic M2 (4MQS) and GPR40 (5TZR) structures in Table 3), not occupied by any ligand. This result has three implications. First, since FTMap has been developed for the analysis of soluble globular proteins, the good performance in finding sites in the intrahelical region suggests that this region is likely to be well solvated. Second, the sites capable of binding allosteric modulators are at least partially formed in structures co-crystallized with only orthosteric modulators. Third, results confirm that the sites capable of binding allosteric ligands in the intrahelical region are among the strongest binding sites, and hence can be detected by energy-based site finding algorithms such as FTMap. However, for a number of sites that bind high affinity ligands the main hot spot at the site includes substantially fewer probe cluster than the number 16 that was required for the high affinity binding in soluble proteins^51^. Thus, the recognition mechanism is still different, and the druggability conditions cannot be automatically extended. Nevertheless, FTMap and FTSite can be considered as useful tools predicting potential allosteric sites in the intrahelical regions of GPCRs. Parametrization and potential other modifications of the method for improving the detection of allosteric sites in the membrane-protein interface requires further development that will be reported in due course.

## Methods

### Mapping the X-ray structures of GPCRs by FTMap and FTSite

The FTMap (http://ftmap.bu.edu/) and FTSite (http://ftsite.bu.edu/) servers were used to map the x-ray structures of each GPCR^38^. Structures were mapped as single chains and also as a single domain. Structures that had missing amino acid side chains were rebuilt using Pymol with the correct residue. This was done to prevent the probes from accessing that region of the protein.

Mapping results were superimposed over the crystal structures and the distance between ligands and predicted sites were calculated. Hot spots and sites within 5 Å of the ligand were considered to correctly identify the binding site.

### Domain splitting

The domains were determined by the Protein Domain Parser algorithm^76^. The algorithm was used to extract the 7TM domain. The 7TM domains were then mapped to ensure that the probes were focused on the relevant domain.

### Analysis of binding pocket by Fpocket

X-ray structures of the orthosteric and allosteric pairs of GPCR complexes were mapped with Fpocket using the default settings^25,26^. The resulting pockets were then compared to the positions of known orthosteric and allosteric ligands. Pockets within 5 Å of the ligand were considered to successfully identify the site.

## Supplementary information

Supplementary material

## Data Availability

The datasets analysed during the current study are available from the corresponding author on reasonable request.

## References

[1] Hauser AS, Attwood MM, Rask-Andersen M, Schioth HB, Gloriam DE (2017). Trends in GPCR drug discovery: new agents, targets and indications. Nat. Rev. Drug. Discov..

[2] Wenthur CJ, Gentry PR, Mathews TP, Lindsley CW (2014). Drugs for allosteric sites on receptors. Annu. Rev. Pharmacol. Toxicol..

[3] Bartuzi D, Kaczor AA, Matosiuk D (2018). Opportunities and Challenges in the Discovery of Allosteric Modulators of GPCRs. Methods Mol. Biol..

[4] Gentry PR, Sexton PM, Christopoulos A (2015). Novel Allosteric Modulators of G Protein-coupled Receptors. J. Biol. Chem..

[5] Smith JS, Lefkowitz RJ, Rajagopal S (2018). Biased signalling: from simple switches to allosteric microprocessors. Nat. Rev. Drug. Discov..

[6] Thal DM, Glukhova A, Sexton PM, Christopoulos A (2018). Structural insights into G-protein-coupled receptor allostery. Nature.

[7] Harrington PE, Fotsch C (2007). Calcium sensing receptor activators: calcimimetics. Curr. Med. Chem..

[8] Dorr P (2005). Maraviroc (UK-427,857), a potent, orally bioavailable, and selective small-molecule inhibitor of chemokine receptor CCR5 with broad-spectrum anti-human immunodeficiency virus type 1 activity. Antimicrob Agents Chemother..

[9] Safdari HA, Pandey S, Shukla AK, Dutta S (2018). Illuminating GPCR Signaling by Cryo-EM. Trends Cell Biol..

[10] Shimada, I., Ueda, T., Kofuku, Y., Eddy, M. T. & Wuthrich, K. GPCR drug discovery: integrating solution NMR data with crystal and cryo-EM structures. *Nat. Rev. Drug. Discov* (2018). 10.1038/nrd.2018.180 PMC6681916 30410121

[11] Congreve M, Oswald C, Marshall FH (2017). Applying Structure-Based Drug Design Approaches to Allosteric Modulators of GPCRs. Trends Pharmacol. Sci..

[12] Robertson N (2011). The properties of thermostabilised G protein-coupled receptors (StaRs) and their use in drug discovery. Neuropharmacology.

[13] Thorsen TS, Matt R, Weis WI, Kobilka BK (2014). Modified T4 Lysozyme Fusion Proteins Facilitate G Protein-Coupled Receptor Crystallogenesis. Structure.

[14] Manglik A, Kobilka BK, Steyaert J (2017). Nanobodies to Study G Protein-Coupled Receptor Structure and Function. Annu. Rev. Pharmacol. Toxicol..

[15] Johnstone S, Albert JS (2017). Pharmacological property optimization for allosteric ligands: A medicinal chemistry perspective. Bioorg. Med. Chem. Lett..

[16] Conn PJ, Kuduk SD, Doller D (2012). Drug Design Strategies for GPCR Allosteric Modulators. Annu. Rep. Med. Chem..

[17] Laskowski RA, Gerick F, Thornton JM (2009). The structural basis of allosteric regulation in proteins. FEBS Lett..

[18] Gunasekaran K, Ma BY, Nussinov R (2004). Is allostery an intrinsic property of all dynamic proteins?. Proteins.

[19] Hardy JA, Wells JA (2004). Searching for new allosteric sites in enzymes. Curr. Opin. Struct. Biol..

[20] Hardy JA, Wells JA (2009). Dissecting an allosteric switch in caspase-7 using chemical and mutational probes. J. Biol. Chem..

[21] Pargellis C (2002). Inhibition of p38 MAP kinase by utilizing a novel allosteric binding site. Nat. Struct. Biol..

[22] Christopoulos A (2002). Allosteric binding sites on cell-surface receptors: novel targets for drug discovery. Nat. Rev. Drug. Discov..

[23] Surade S (2014). A structure-guided fragment-based approach for the discovery of allosteric inhibitors targeting the lipophilic binding site of transcription factor EthR. Biochem. J..

[24] Huang W (2013). Allosite: a method for predicting allosteric sites. Bioinformatics.

[25] Le Guilloux V, Schmidtke P, Tuffery P (2009). Fpocket: an open source platform for ligand pocket detection. BMC Bioinformatics.

[26] Schmidtke P, Le Guilloux V, Maupetit J, Tuffery P (2010). fpocket: online tools for protein ensemble pocket detection and tracking. Nucleic Acids Res..

[27] Huang B, Schroeder M (2006). LIGSITEcsc: predicting ligand binding sites using the Connolly surface and degree of conservation. BMC Struct. Biol..

[28] Greener JG, Filippis I, Sternberg MJE (2017). Predicting Protein Dynamics and Allostery Using Multi-Protein Atomic Distance Constraints. Structure.

[29] Huang M (2018). AlloFinder: a strategy for allosteric modulator discovery and allosterome analyses. Nucleic Acids Res..

[30] Goodford PJ (1985). A computational procedure for determining energetically favorable binding sites on biologically important macromolecules. J. Med. Chem..

[31] Halgren TA (2009). Identifying and Characterizing Binding Sites and Assessing Druggability. J. Chem. Inf. Model..

[32] Ghanakota P, Carlson HA (2016). Moving Beyond Active-Site Detection: MixMD Applied to Allosteric Systems. J. Phys. Chem. B.

[33] Miao Y, Nichols SE, McCammon JA (2014). Mapping of allosteric druggable sites in activation-associated conformers of the M2 muscarinic receptor. Chem. Biol. Drug. Des..

[34] Ivetac A, McCammon JA (2012). A molecular dynamics ensemble-based approach for the mapping of druggable binding sites. Methods Mol. Biol..

[35] Caliman AD, Miao Y, McCammon JA (2018). Mapping the allosteric sites of the A2A adenosine receptor. Chem. Biol. Drug. Des..

[36] Ivetac A, McCammon JA (2010). Mapping the druggable allosteric space of G-protein coupled receptors: a fragment-based molecular dynamics approach. Chem. Biol. Drug. Des..

[37] Brenke R (2009). Fragment-based identification of druggable ‘hot spots’ of proteins using Fourier domain correlation techniques. Bioinformatics.

[38] Kozakov D (2015). The FTMap family of web servers for determining and characterizing ligand-binding hot spots of proteins. Nat. Protoc..

[39] DeLano WL (2002). Unraveling hot spots in binding interfaces: progress and challenges. Curr. Opin. Struct. Biol..

[40] Ciulli A, Williams G, Smith AG, Blundell TL, Abell C (2006). Probing hot spots at protein-ligand binding sites: A fragment-based approach using biophysical methods. J. Med. Chem..

[41] Metz A (2012). Hot Spots and Transient Pockets: Predicting the Determinants of Small-Molecule Binding to a Protein-Protein Interface. J. Chem. Inf. Model..

[42] Hall DR, Kozakov D, Whitty A, Vajda S (2015). Lessons from Hot Spot Analysis for Fragment-Based Drug Discovery. Trends Pharmacol. Sci..

[43] Dennis S, Kortvelyesi T, Vajda S (2002). Computational mapping identifies the binding sites of organic solvents on proteins. Proc. Natl. Acad. Sci. USA.

[44] Landon MR, Lancia DR, Yu J, Thiel SC, Vajda S (2007). Identification of hot spots within druggable binding regions by computational solvent mapping of proteins. J. Med. Chem..

[45] Landon MR (2009). Detection of ligand binding hot spots on protein surfaces via fragment-based methods: application to DJ-1 and glucocerebrosidase. J. Comput. Aid. Mol. Des..

[46] Chuang GY (2009). Binding hot spots and amantadine orientation in the influenza a virus M2 proton channel. Biophys. J..

[47] Buhrman G (2011). Analysis of binding site hot spots on the surface of Ras GTPase. J. Mol. Biol..

[48] Zerbe BS, Hall DR, Vajda S, Whitty A, Kozakov D (2012). Relationship between Hot Spot Residues and Ligand Binding Hot Spots in Protein-Protein Interfaces. J. Chem. Inf. Model..

[49] Golden MS (2013). Comprehensive experimental and computational analysis of binding energy hot spots at the NF-kappaB essential modulator/IKKbeta protein-protein interface. J. Am. Chem. Soc..

[50] Ngan CH (2012). FTSite: high accuracy detection of ligand binding sites on unbound protein structures. Bioinformatics.

[51] Kozakov D (2015). New Frontiers in Druggability. J Med Chem.

[52] Kozakov D, Chuang GY, Beglov D, Vajda S (2010). Where does amantadine bind to the influenza virus M2 proton channel?. Trends Biochem. Sci..

[53] Berman HM (2000). The Protein Data Bank. Nucleic Acids Res..

[54] Kruse AC (2013). Activation and allosteric modulation of a muscarinic acetylcholine receptor. Nature.

[55] Wu B (2010). Structures of the CXCR4 chemokine GPCR with small-molecule and cyclic peptide antagonists. Science.

[56] Tan Q (2013). Structure of the CCR5 chemokine receptor-HIV entry inhibitor maraviroc complex. Science.

[57] Wu H (2014). Structure of a class C GPCR metabotropic glutamate receptor 1 bound to an allosteric modulator. Science.

[58] Christopher JA (2015). Fragment and Structure-Based Drug Discovery for a Class C GPCR: Discovery of the mGlu5 Negative Allosteric Modulator HTL14242 (3-Chloro-5-[6-(5-fluoropyridin-2-yl)pyrimidin-4-yl]benzonitrile). J. Med. Chem..

[59] Dore AS (2014). Structure of class C GPCR metabotropic glutamate receptor 5 transmembrane domain. Nature.

[60] Christopher, J. A. *et al*. Structure-Based Optimization Strategies for G Protein-Coupled Receptor (GPCR) Allosteric Modulators: A Case Study from Analyses of New Metabotropic Glutamate Receptor 5 (mGlu5) X-ray Structures. *J. Med. Chem*. (2018). 10.1021/acs.jmedchem.7b01722 29455526

[61] Byrne EFX (2016). Structural basis of Smoothened regulation by its extracellular domains. Nature.

[62] Wang C (2014). Structural basis for Smoothened receptor modulation and chemoresistance to anticancer drugs. Nature Comm..

[63] Cheng RKY (2017). Structural insight into allosteric modulation of protease-activated receptor 2. Nature.

[64] Hollenstein K (2013). Structure of class B GPCR corticotropin-releasing factor receptor 1. Nature.

[65] Zhang D (2015). Two disparate ligand-binding sites in the human P2Y1 receptor. Nature.

[66] Robertson N (2018). Structure of the complement C5a receptor bound to the extra-helical antagonist NDT9513727. Nature.

[67] Jazayeri A (2016). Extra-helical binding site of a glucagon receptor antagonist. Nature.

[68] Zhang H (2017). Structure of the full-length glucagon class B G-protein-coupled receptor. Nature.

[69] Srivastava A (2014). High-resolution structure of the human GPR40 receptor bound to allosteric agonist TAK-875. Nature.

[70] Lu J (2017). Structural basis for the cooperative allosteric activation of the free fatty acid receptor GPR40. Nat. Struct. Mol. Biol..

[71] Zheng Y (2016). Structure of CC chemokine receptor 2 with orthosteric and allosteric antagonists. Nature.

[72] Liu XY (2017). Mechanism of intracellular allosteric beta(2)AR antagonist revealed by X-ray crystal structure. Nature.

[73] Oswald C (2016). Intracellular allosteric antagonism of the CCR9 receptor. Nature.

[74] Song GJ (2017). Human GLP-1 receptor transmembrane domain structure in complex with allosteric modulators. Nature.

[75] Cherezov V (2007). High-resolution crystal structure of an engineered human beta(2)-adrenergic G protein-coupled receptor. Science.

[76] Alexandrov N, Shindyalov I (2003). PDP: protein domain parser. Bioinformatics.

