# Supplementary material for: Analysis of tractable allosteric sites in G protein-coupled receptors
Source: Sci Rep. 2019 Apr 16;9:6180. doi: 10.1038/s41598-019-42618-8 (PMC6467999; doi:10.1038/s41598-019-42618-8)

Supporting Information

Analysis of tractable allosteric sites in G protein-coupled receptors

Amanda Wakefield[1,2], Jonathan S Mason[3], Sandor Vajda[1,2]* and György M. Keserű[4]*

[1]Department of Chemistry, Boston University, Boston, MA 02215, USA

[2]Department of Biomedical Engineering, Boston University, Boston, MA 02215, USA

[3]Sosei Heptares, Steinmetz Building, Granta Park, Great Abington, Cambridge CB21 6DG., U.K.

[4]Medicinal Chemistry Research Group, Research Center for Natural Sciences, Magyar tudósok krt. 2. H-1117 Budapest, Hungary

Corresponding Author

* György Miklós Keserű: keseru.gyorgy@ttk.mta.hu

Figure S1. Hot spots and ligand binding sites predicted, respectively, by (a) FTmap and (b) FTSite for the MGLU5-CMPD-25 (PDB:5CGC). The allosteric ligand CMPD-25 is represented by green sticks. The FTMap hot spot 5(7) is shown in orange. The third ranked site predicted by FTSite, represented as purple mesh, overlapped with the ligand binding site.

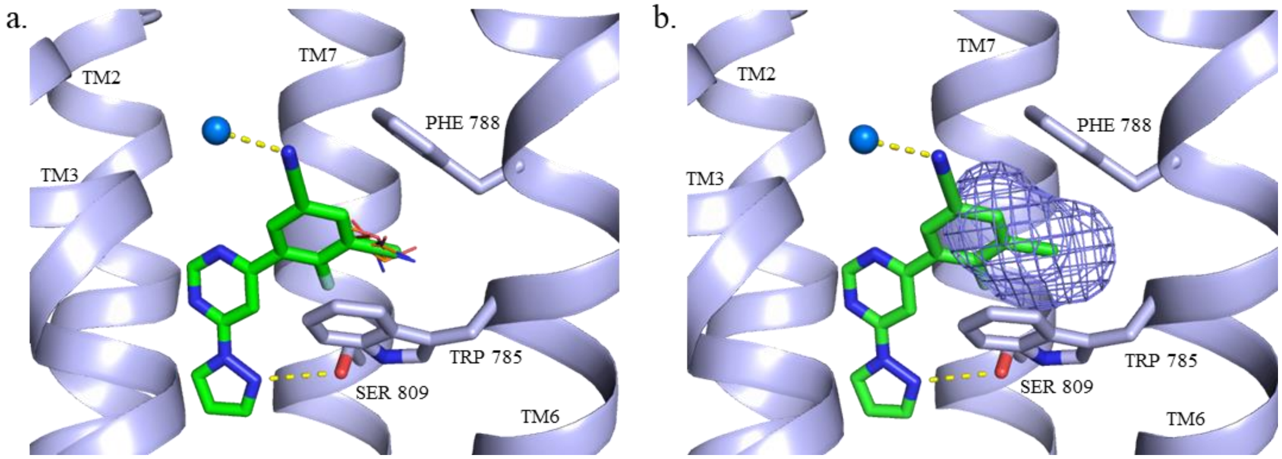

Table S1. Characterization of the ligand binding sites in orthosteric and allosteric pairs of GPCR complexes by FPocket

Structure ID	Pockets identified	Orthosteric site volume	Allosteric site volume	Orthosteric site druggability	Allosteric site druggability
**2RH1**	Pocket 1 overlaps with the CAU orthosteric site, pocket 2 overlaps with the allosteric ligand site	2871.183	1306.119	0.751	0.3
**5X7D**	Pocket 1 overlaps with the CAU orthosteric site, pockets 2 and 11 overlap with the allosteric ligand site	1486.681	1325.743 + 431.368	0.544	0.416 + 0.015
**4MQS**	Pocket 3 overlaps with the IX0 orthosteric site. Pocket 2 overlaps with the 2CU allosteric ligand site.	411.602	1308.812	0.521	0.062
**4MQT**	Pocket 1 overlaps with both the orthosteric and allosteric sites.	1808.916		0.795	
**5TZR**	Pocket 1 overlaps with the orthosteric MK6 site. Pocket 16 overlaps with the 70S allosteric site.	531.095	584.646	0.908	0.159
**5TZY**	Pocket 1 overlaps with the orthosteric MK6 site. Pocket 5 overlaps with the 70S allosteric site.	715.106	1207.94	0.812	0.267
**4XNW**	Pocket 1 overlaps with the orthosteric 2ID site. Pocket 10 overlaps with the BUR allosteric site.	1160.696	522.432	0.162	0.198
**4XNV**	No pocket overlaps with BUR. The orthosteric site is unbound but the 2ID ligand from 4XNW overlaps with pocket 1	1798.08	N/A	0.306	N/A

Figure S2. Hot spots and ligand binding sites predicted, respectively, by (a) FTmap and (b) FTSite for the mGluR5-M-MPEP structure (PDB: 6FFI). The allosteric ligand M-MPEP is represented by green sticks. The FTMap hot spots, shown as lines, are 1(17) shown in pink and 5(5) shown in blue. The sites predicted by FTSite did not overlap with the ligand binding site.

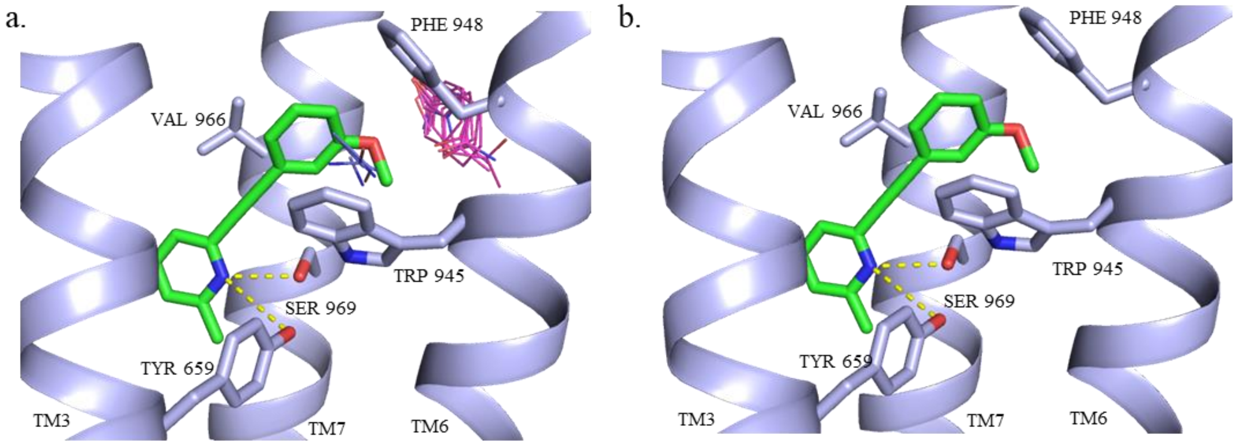

Figure S3. Hot spots and ligand binding sites predicted, respectively, by (a) FTmap and (b) FTSite for the mGluR5-fenobam structure (PDB: 6FFH). The allosteric ligand fenobam is represented by green sticks. The FTMap hot spots, 3(10) shown in light pink lines and 7(5) shown in purple lines. The sites predicted by FTSite did not overlap with the ligand binding site.

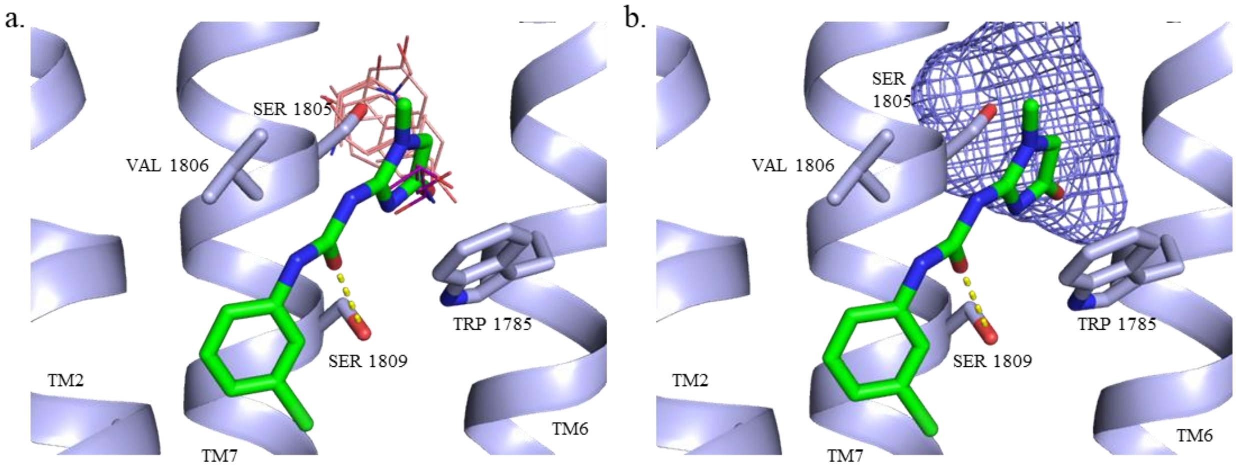

Supplement: Supplementary file 1 — Supplementary material [file 41598_2019_42618_MOESM1_ESM.pdf]
